# Exploring the dynamics and influencing factors of CD4 T cell activation using single-cell RNA-seq

**DOI:** 10.1016/j.isci.2023.107588

**Published:** 2023-08-09

**Authors:** Hui Li, Hongyi Liu, Yifei Liu, Xuefei Wang, Shiya Yu, Hongwen Huang, Xiangru Shen, Qi Zhang, Ni Hong, Wenfei Jin

**Affiliations:** 1 School of Life Sciences, Southern University of Science and Technology, Shenzhen 518055, China; 2 CAS Key Laboratory of Computational Biology, Shanghai Institute of Nutrition and Health, University of Chinese Academy of Sciences, Chinese Academy of Sciences, Shanghai 200031, China

**Keywords:** Immune response, Components of the immune system, Specialized functions of cells, Transcriptomics

## Abstract

T cell activation is a key event in adaptive immunity. However, the dynamics and influencing factors of T cell activation remain unclear. Here, we analyzed CD4 T cells that were stimulated with anti-CD3/CD28 under several conditions to explore the factors affecting T cell activation. We found a stimulated T subset (HSP^hi^ T) highly expressing heat shock proteins, which was derived from stimulated naive T. We identified and characterized inert T, a stimulated T cell subset in transitional state from resting T to activated T. Interestingly, resting *CXCR4*^low^ T responded to stimulation more efficiently than resting *CXCR4*^hi^ T. Furthermore, stimulation of CD4 T in the presence of CD8 T resulted in more effector T and more homogeneous expressions of CD25, supporting that presence of CD8 T reduces the extreme response of T cells, which can be explained by regulation of CD4 T activation through CD8 T-initiated cytokine signaling and FAS/FASLG signaling.

## Highlights

• Inert T represents a transitional state of stimulated T cells
• The HSP^hi^ T is primarily derived from stimulated naive T cells
• CD8 T significantly reduced the HSP^hi^ and IFN^hi^ T cell subsets
• Resting CXCR4^low^ T responded efficiently to stimulation

## Graphical abstract

**Figure:**
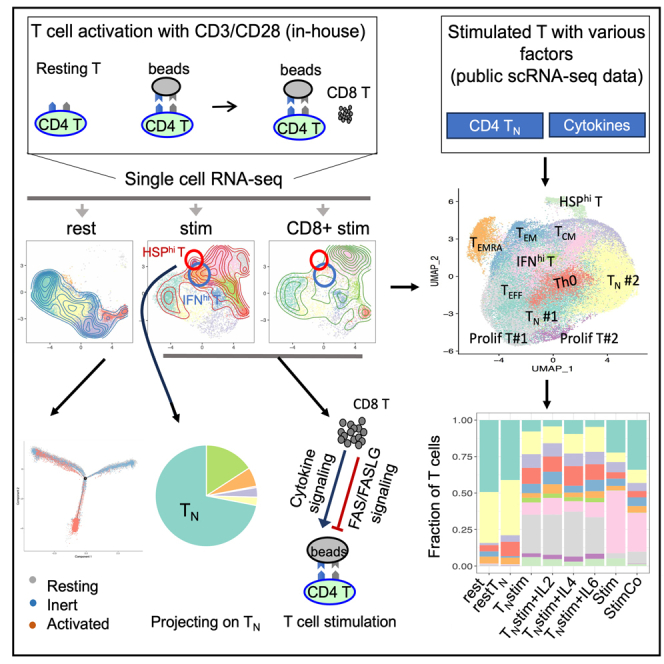

## Introduction

CD4 T cells are a key component of adaptive immunity, playing important roles not only in pathogens clearance but also in regulation of autoimmune disease and clearance of pathogenic cells such as cancer cells.^1^^,^^2^^,^^3^^,^^4^^,^^5^^,^^6^ If T cell receptor (TCR) on the surface of T cells recognizes an antigen-loaded major histocompatibility complex (MHC), the T cells will quickly differentiate and proliferate into a large number of effector T cells (T_EFF_) that recognize the same antigen, which is called T cell activation.^7^^,^^8^^,^^9^ T cell activation is crucial in establishing and controlling adaptive immune responses against pathogens and pathogenic cells and is the core of adaptive immune response.^10^^,^^11^ The most commonly used model to study CD4 T cell activation is the stimulation of naive CD4 T cells (T_N_) with anti-CD3/CD28-coated beads. CD4 T_N_, which egress from thymus before they encounter any antigen, are fairly quiescent and can differentiate into different subtypes of T_EFF_ after stimulation. Studies of T cell activation based on CD4 T_N_ have significantly increased our understanding of T cell activation process.^12^^,^^13^^,^^14^^,^^15^ In particular, different cytokines polarized CD4 T_N_ into different T_EFF_ subsets; e.g., CD4 T_N_ could be polarized by interferon (IFN)-γ, interleukin (IL)-4, and IL-17 into Th1, Th2, and Th17, respectively.^16^^,^^17^^,^^18^^,^^19^

Different from relatively homogeneous CD4 T_N_, CD4 T cells in human peripheral blood are a heterogeneous cell population including several T cell subsets including T_N_, central memory T cells (T_CM_), effector memory T cells (T_EM_), and regulatory T cells (Treg).^4^^,^^20^^,^^21^^,^^22^ Different CD4 T cell subsets have different responses to stimulation. For example, T_CM_ and T_EM_ quickly proliferate and secrete cytokines upon stimulation, while T_N_ responds to stimulation much slowly and could differentiate into various T cell subsets under different microenvironment.^8^^,^^23^^,^^24^^,^^25^ The development of single-cell RNA sequencing (scRNA-seq) provides a unique opportunity to investigate the effects of multiple factors on T cell activation. Bibby et al.^26^ developed a single-cell pathway analysis tool and applied it to characterize pathways that are critical for human T cell activation. Andreatta et al.^27^ generated reference T cell atlases to interpret T cell states under cancer and viral infection. Zhang et al.^28^ applied scRNA-seq and single-cell Assay for Transposase-Accessible Chromatin with high-throughput sequencing (scATAC-seq) to explore the mechanism underlying immune aging-induced low-inflammatory state in naive CD4 T cells. Recently, Cano-Gamez et al.^20^ analyzed CD4 memory T cell (T_M_) and showed that T cell activation is influenced by effectorness gradient. CD4 T_N_ differentiates into Th2 with IL-4 polarization, while CD4 T_M_ could not differentiate into Th2 phenotype. Soskic et al. identified lots of genetic variants that regulate gene expression dynamics during CD4 T cell activation.^29^ However, many factors affecting T cell activation have not been explored. In particular, there is no systematic study on how cell heterogeneity and T cell communications affect T cell activation.

Here, we stimulated peripheral CD4 T cells with anti-CD3/CD28 beads and performed scRNA-seq to investigate the effects of stimulations on CD4 T cell activation. Integrated analyses of CD4 T cells pre- and post-stimulation identified distinct CD4 T cell subsets, including T_N_, T_CM_, T_EM_, Treg, effector memory T cells re-expressing CD45RA (T_EMRA_), 4 different T_EFF_ subsets, and HSP^hi^ T. We identified two main trajectories of T cell activation both starting from T_N_ and progressing to T_EFF_, with differences lying in whether they pass through T_M_. We called the stimulated T cells that overlapped with resting T cells on uniform manifold approximation and projection (UMAP) as inert T. Compared with resting T, inert T highly expressed nuclear factor κB (NF-κB) signaling pathway and its target genes. Through a series of comparisons of the dynamics and signals of CD4 T cell subsets under various stimulations, we found that CD4 T cell subsets, cell-cell communications, and cytokines shaped activated T subsets, gene expression patterns, and cell states.

## Results

### T cell subsets pre- and post-CD4^+^ T cell activation

**Figure 1:**
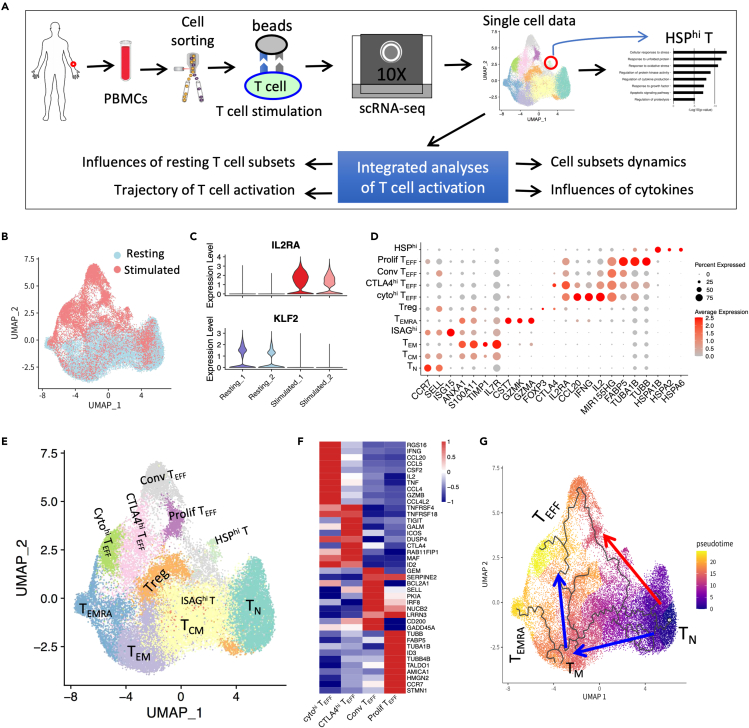
Scheme of this study and T cell subsets pre- and post-stimulation (A) Scheme of this study. (B) UMAP projection of CD4 T cells pre- and post-*anti*-CD3/CD28 stimulation, colored by whether it was stimulated. (C) Expression level of *IL2RA* and *KLF2* in resting T cells and stimulated T cells. IL2RA is activated T marker, while KLF2 is naive T marker. (D) Normalized expression level and expression percentage of cell-type-specific genes in 11 T cell subsets. Also see Data S1. (E) UMAP projection of CD4 T cells pre- and post-*anti*-CD3/CD28 stimulation, colored by cell subsets. (F) Heatmap of expression level of DEGs between conv T_EFF_, CTLAA4^hi^ T_EFF_, cyto^hi^ T_EFF_, and prolif T_EFF_. (G) Two major T cell activation trajectories inferred by Monocle 3, colored by cell’s pseudotime value. Also see Figure S1.

We designed a workflow to analyze dynamics/signals of T cell subsets during T cell activation and the influencing factors based on anti-CD3/CD28 stimulation of CD4 T cells (Figure 1A). In brief, we isolated peripheral CD4 T cells by CD4 MicroBeads from a healthy donor. The peripheral CD4 T cells were stimulated with anti-CD3/CD28 magnetic beads (Dynabeads Human T-Activator CD3/CD28 for T cell Expansion and Activation) for 16 h. We performed scRNA-seq on both resting peripheral CD4 T cells and stimulated CD4 T cells using 10x Genomics Chromium Single Cell 3′ Library & Gel Bead Kit v2. A total of 31,815 CD4 T cells, including 21,573 resting CD4 T cells and 10,242 stimulated CD4 T cells, passed quality control. UMAP plot showed that resting peripheral CD4 T cells and stimulated CD4 T cells displayed quite different distributions (Figure 1B). The resting CD4 T cells were concentrated in the lower part of the UMAP plot, while the stimulated CD4 T cells were widely distributed, with majority in the upper part (Figure 1B). Indeed, the expression levels of activated T cell marker *IL2RA*^30^ and naive T cell marker *KLF2*^31^ were upregulated and downregulated after stimulation, respectively (Figure 1C). We identified 69 genes specifically expressed in resting T cells and 253 genes specifically expressed in stimulated T cells (figures S1A and S1B), with majority of these findings being consistent with our previous study using bulk data.^32^

We identified 11 T cell subsets and annotated them according to their specific expressed genes (Figures 1D and 1E). There are 6 T cell subsets mainly from resting CD4 T cells, namely T_N_ (*CCR7*^hi^*, SELL*^hi^), T_CM_ (*CCR7*^+^*, SELL*^+^, *ANXA1*^+^), T_EM_ (*CCR7*^*low*^, ANXA1^+^, *TIMP1*^*+*^), CD4 T_EMRA_ (*ANXA1*^+^*, CST7*^*+*^*, GZMK*^*+*^, *GZMA*^*+*^), Treg (*FOXP3*^+^, *CTLA4*^+^), and IFN signaling-associated gene (ISAG) highly expressed T cells (ISAG^hi^ T) (*ISG15*^hi^*, CCR7*^*+*^, *SELL*^+^) (Figures 1D, 1E, S1C, and S1D). These T cell subsets are essentially consistent with recent studies on peripheral CD4 T cells.^4^^,^^20^^,^^29^ There are 5 T cell subsets mainly from stimulated CD4 T cells, among which there is a small T cell subset that specifically expresses heat shock proteins (HSPs), such as *HSPB1, HSPA1A*, and *HSPA6* (Figure 1D and Data S1), and is called HSP^hi^ T. The other four T cell subsets mainly from stimulated CD4 T cells belong to CD4 T_EFF_ (*IL2RA*^+^*, IL2*^+^*, NME1*^+^, *MIR155HG*^+^*,* and *FABP5*^+^) (Figures 1D and 1E). We further found that each of the four T_EFF_ subsets has special features and called them conventional T_EFF_ (conv T_EFF_), cytokines high T_EFF_ (cyto^hi^) T_EFF_, proliferation T_EFF_ (prolif T_EFF_), and CTLA4^hi^ T_EFF_ (Figures 1D, 1F, S1E, and S1F).

We inferred the pseudotime trajectories of these T cells for better understanding of T cell activation processes using Monocle 3. The inferred pseudotime contained two major trajectories: 1) T_N_ → T_EFF_ and 2) T_N_ → T_CM_ → T_EM_ → T_EFF_. Both trajectories started from T_N_ and ended at T_EFF_, with different intermediate processes. The first trajectory initiated at T_N_ and directly differentiated into T_EFF_, which is short and direct T cell activation process. The second trajectory has two main differentiation phases: phase #1 refers to the differentiation of T_N_ into T_M_, and phase #2 refers to the differentiation of T_M_ into T_EFF_ (Figure 1G). Monocle2 analysis showed T_N_ and T_EM_/T_EMRA_ mainly located on two different branches and finally differentiated into T_EFF_ (figures S2A and S2B), which is essentially consistent with the two inferred trajectories by monoclye3. Furthermore, the expressions of activated T-specific genes (*MIR155HG*, *IER3*) and cytotoxicity-related genes (*NKG7*, *GZMH*) were upregulated alongside the trajectory (Figure S2C).

### Feature and origin of HSP^hi^ T

**Figure 2:**
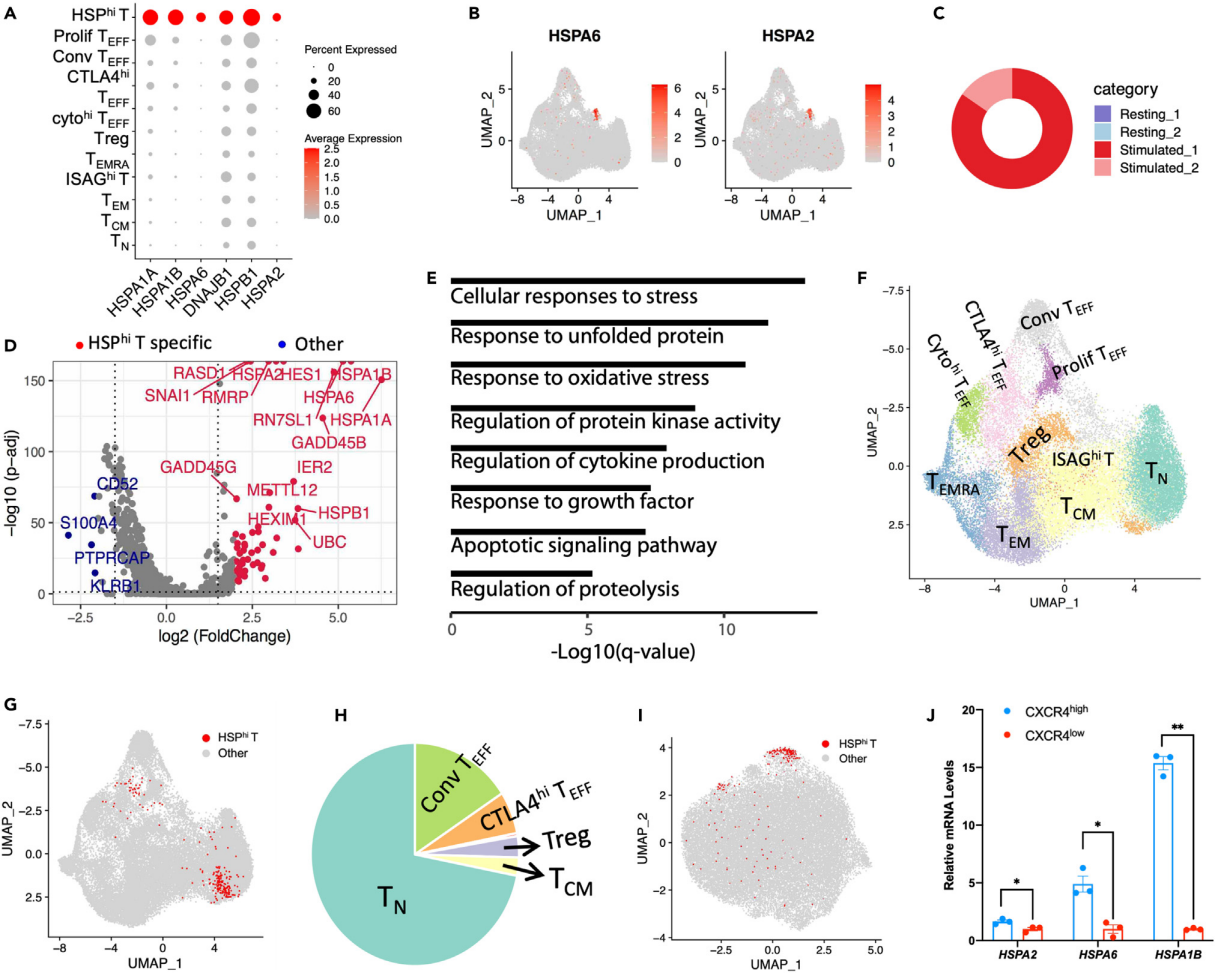
Feature of HSP^hi^ T (A) Dot plot of HSP^hi^ T-specific expressed genes. Color represents normalized expression level, and dot size represents expression percentage. (B) Expression level of *HSPA6* and *HSPA2* on UMAP plot of CD4 T cells. (C) Cell composition of HSP^hi^ T based on cell sources. (D) Volcano plot of DEGs between HSP^hi^ T and the other T cells (p < 0.01). Red points represent HSP^hi^ T cell-specific highly expressed genes, while blue points represent the other T cell-specific highly expressed genes. (E) GO enrichment analysis of HSP^hi^ T-specific expressed genes. (F) UMAP projection of CD4 T cells using gene sets that removed HSP genes, in which HSP^hi^ T cell cluster disappears. (G) UMAP projection of CD4 T cells using gene sets that removed HSP genes, with red points highlighting the original identified HSP^hi^ T cells. (H) Pie plot of number of HSP^hi^ T projected on each CD4 T cell subset, with 72% projected on T_N_. (I) UMAP projection CD4 T cells based on HSP genes, with red points highlighting original identified HSP^hi^ T cells. (J) Expression of HSP genes in FACS-enriched HSP^hi^ T (CXCR4^high^ T) and CXCR4^low^ cells, measured by qPCR. Also see Figure S2. Data were shown as mean ± SEM.

HSP^hi^ T is a small T cell subset that specifically expresses HSP genes such as *HSPA6*, *HSPA1A*, and *HSPA1B* (Figures 2A and 2B). HSP^hi^ T were solely from stimulated CD4 T cells, without contribution from resting T cells (Figure 2C), indicating HSP^hi^ T is a specific state of stimulated T. Comparing with other T cells, HSP^hi^ T specifically expressed *HSPA1B*, *HSPA1A*, *HSPA2*, *HSPA6, METTL12, GADD45B, IER2, IER*, and *DNAJB1* (Figure 2D). These HSP^hi^ T-specific genes-enriched Gene Ontology (GO) terms were cellular responses to stress (p = 1.3 × 10^−13^), response to unfolded protein (p = 2.5 × 10^−12^), response to oxidative stress (p = 1.7 × 10^−11^), regulation of protein kinase activity (p = 1.0 × 10^−9^), regulation of cytokine production (p = 1.1 × 10^−8^), apoptotic signaling pathway (p = 6.4 × 10^−8^), and regulation of proteolysis (p = 6.2 × 10^−6^) (Figure 2E). Based on the HSP^hi^ T-specific enriched GO, HSP^hi^ T might be derived from T cells that over-responded to anti-CD3/CD28 beads stimulation.

We re-conducted dimension reduction and clustering analysis on the CD4 T cells using gene set that excluded HSP genes. The re-clustered T cell subsets were very similar to those based on genome-wide data, except for the disappearance of HSP^hi^ T cluster (Figure 2F). On the UMAP plot based on gene set that excluded HSP genes, HSP^hi^ T was mainly projected on T_N_ (71.93%), with moderate projection on conv T_EFF_ (15.78%) and a small fraction on *CTLA4*^hi^ T_EFF_ (6.14%), Treg (3.1%), and cyto^hi^ T_EFF_ (0.43%) (Figures 2G and 2H). After normalizing the cell number of HSP^hi^ T to its projected T cell subset, the percentage of HSP^hi^ T in CD4 T_N_ was still the highest among all T cell subsets (Figure S2D). The majority of HSP^hi^ T being projected onto CD4 T_N_ indicates that the gene expression profile of HSP^hi^ T has the highest similarity to that of CD4 T_N_ when HSP genes are ignored. These results further indicate that majority of HSP^hi^ T were derived from CD4 T_N_ that respond to anti-CD3/CD28 stimulation. Interestingly, HSP^hi^ T cluster showed up when we only used HSP genes to conduct UMAP (Figure 2I), further indicating that HSP genes were the major contributor for the formation of HSP^hi^ T cluster. We enriched HSP^hi^ T by sorting out cells with high expression of CXC chemokine receptor subtype 4 (*CXCR4)* from stimulated CD4 T cells since HSP^hi^ T expressed the highest *CXCR4* among all stimulated T cell subsets (figures S2E and S2F). qPCR showed the enriched HSP^hi^ T indeed highly expressed HSP genes such as *HSPA2*, *HSPA6*, and *HSPA1B* (Figure 2J).

### Feature of inert T

**Figure 3:**
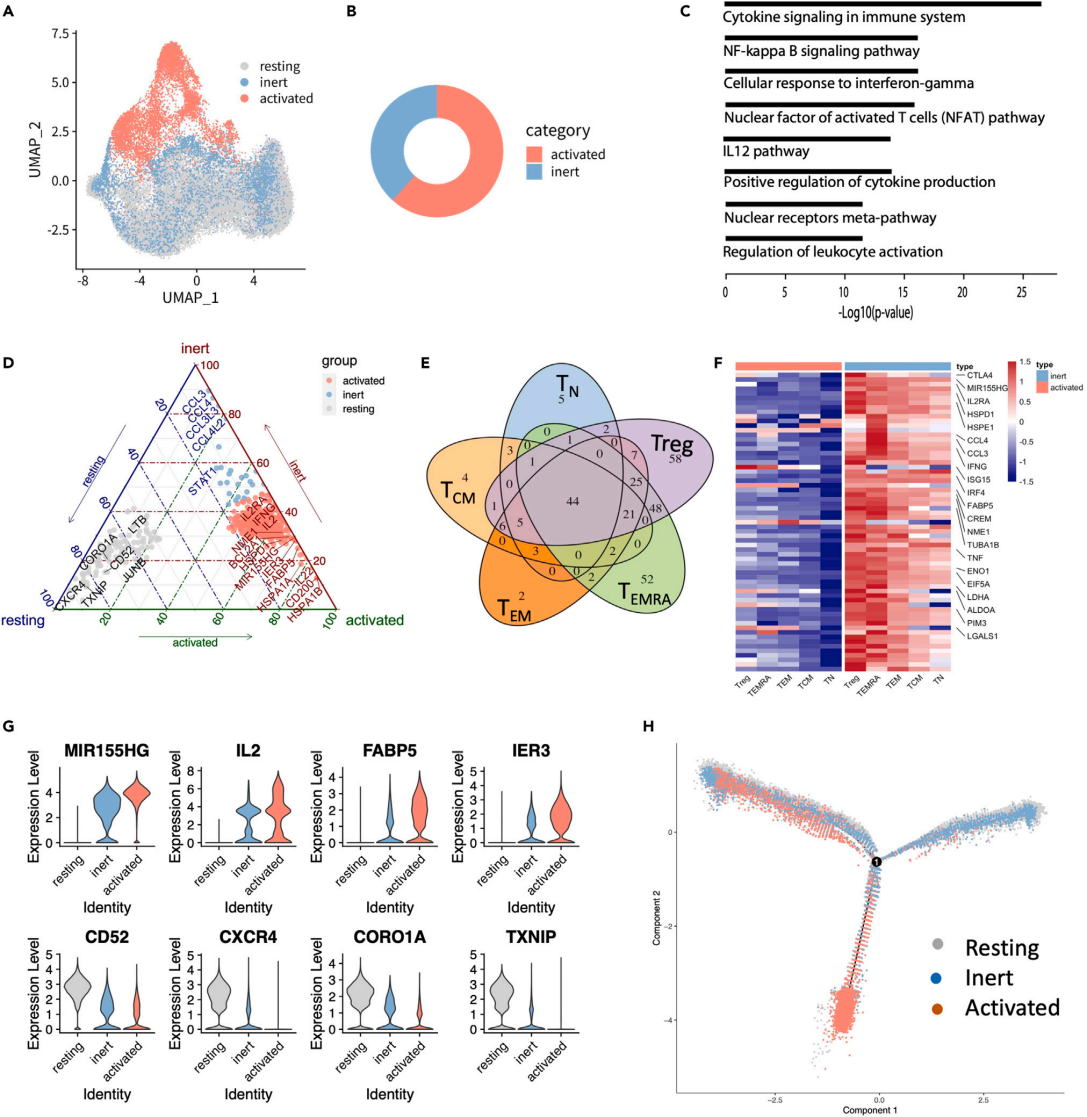
Feature of inert T (A) UMAP projection of CD4 T cells, colored by resting T, inert T, and activated T. (B) The proportion of inert T and activated T cells to the total stimulated T cells. (C) GO analysis of inert T subset-specific genes compared with resting T cells. Also see Data S2. (D) Ternary diagram of T cell subsets-specific genes between resting T, inert T, and activated T. Also see Data S3. (E) Venn diagrams of inert T-specific genes in T_N_, T_CM_, T_EM_, T_EMRA_, and Treg (avg_logFC ≥ 1). The inert T-specific genes were calculated by comparing inert T to resting T in each T cell subset. (F) Gene expression heatmap of inert T-specific genes that shared among T_N_, T_CM_, T_EM_, T_EMRA_, and Treg. (G) Expression level of activated T-specific genes (top) and resting T-specific genes (bottom) in resting T, inert T, and activated T. (H) Ordering of cells along pseudotime in two-dimensional state space defined by Monocle2. Cells are colored by cellular clusters. Also see Figure S3.

The upper part of UMAP plot is stimulated T-specific subsets, including conv T_EFF_, cyto^hi^ T_EFF_, prolif T_EFF_, and HSP^hi^ T, collectively referred to as activated T cells (Figure 3A). On the other hand, stimulated T cells that overlap with the resting T cells on UMAP plot should have similar expression profile to that of resting T cells and thus were called inert T cells (Figure 3A). In this way, the stimulated T cells were divided into 6,316 activated T cells (61.67%) and 3,926 inert T cells (38.33%) (Figure 3B). Compared with resting T, we identified 139 inert T-specific genes and 38 inert low-expressed genes. The 139 inert T-specific genes included activated T-specific genes (*IL2RA*, *NME1*, *MIR155HG*, *FABP5, IL2, CD200*, and *IER3*) and cytokine genes (*CCL3*, *CCL4*, *CCL3L3*, *CCL4L2*, *IL22, IFNG*, and *STAT1*) (Data S2). Furthermore, these inert T-specific genes significantly enriched in cytokine signaling (3.31 × 10^−27^), NF-κB signaling pathway (p = 5.50 × 10^−17^), cellular response to IFN-γ (p = 5.50 × 10^−17^), Nuclear factor of activated T cells (NFAT) pathway (p = 1.17 × 10^−16^), IL-12 pathway (p = 5.50 × 10^−15^), positive regulation of cytokine production (p = 8.13 × 10^−15^), Nuclear receptors meta-pathway (p = 1.66 × 10^−13^), and regulation of leukocyte activation (p = 3.02 × 10^−12^) (Figure 3C), indicating that inert T has partially responded to the stimulation, despite their overall expression profile being similar to that of resting T cells. Compared with activated T, inert T highly expressed *CCL3*, *CCL4*, *CCL4L2*, *CCL3L3*, *TXNIP*, *CD52*, *CXCR4, CORO1A*, and so on (Data S3), which were enriched in GO terms such as response to stimulus, locomotion, and immune system process and signaling. Compared with both resting T and activated T, inert T specifically expressed cytokine genes such as *CCL3, CCL4, CCL3L3*, and *CCL4L2* (Figure 3D), indicating inert T is not a simple transition state but has its own specific feature.

We further separated each of the five T cell subsets, namely T_N_, T_CM_, T_EM_, T_EMRA_, and Treg, into resting T subset and inert T subset; e.g., T_N_ was separated into resting T_N_ and inert T_N_. The differentially expressed genes (DEGs) between resting T_N_ and inert T_N_ were similar to those between resting T and inert T (Figure S3A). The specifically upregulated/downregulated genes of inert T cell subsets, compared with their resting T cell counterparts, were largely shared (Figures 3E, 3F, and S3B), indicating the features of inert T subsets are essentially consistent. On the other hand, inert T is a specific state with many genes having intermediate expression level between resting T and activated T (Figure 3G). For example, compared with its high expression in resting T cells and its almost silence in activated T cells, *CXCR4* is moderately expressed in inert T cells (Figure 3G). Because *CXCR4* is a key CXC chemokine receptor for mediation of cell migration, its high expression in resting T may facilitate the migration of resting T to its targeted locations.^33^^,^^34^

Fluorescence-activated cell sorting (FACS) analysis of stimulated CD4 T cells showed some T cells that did not express CD25, which could be mainly inert T (Figure S3C). Furthermore, pseudotime analyses showed that inert T differentiated into activated T along the inferred trajectory, similar to resting T (Figure 3H). The activated T increase and inert T decrease after extended stimulations (after another 16 h) (Figures S3D and S3E), supporting that inert T presents a transitional state and could differentiate into activated T following prolonged stimulation.

### CXCR4 level is associated with efficiency of T cell activation

**Figure 4:**
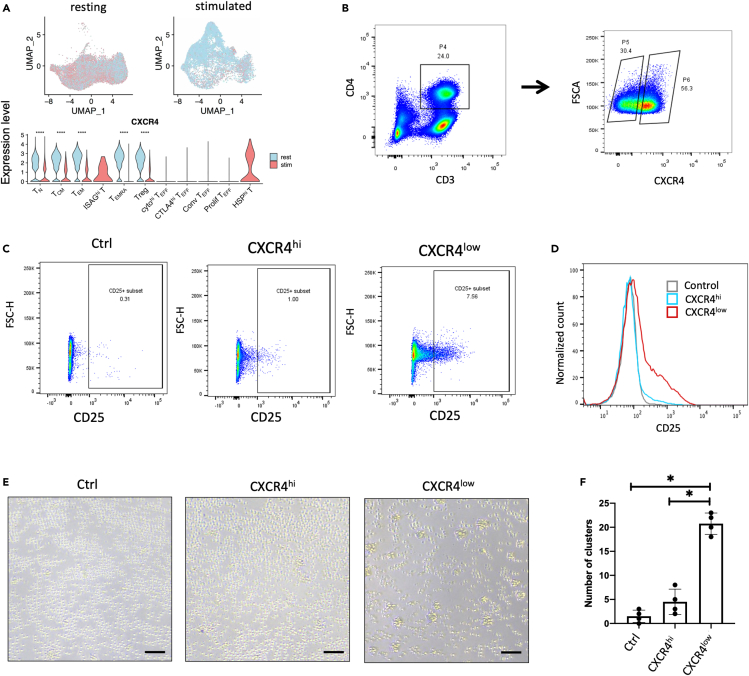
Expression level of *CXCR4* is significantly associated with the efficiency of T cell activation (A) Expression level of *CXCR4* on UMAP projection (top), and in CD4 T cell subsets (bottom) pre- and post-stimulation. (B) Sorting of CXCR4^hi^ CD4 T cells and CXCR4^low^ C4+ T cells from PBMCs by FACS. (C) CD25 level in control (left), anti-CD3/CD28-stimulated CXCR4^low^ CD4 T cells (middle), and anti-CD3/CD28-stimulated CXCR4^hi^ CD4 T cells by FACS sorting (right). (D) Comparison of CD25 level in control, stimulated T derived from *CXCR4*^low^ T, and stimulated T derived from *CXCR4*^hi^ T. (E) Images of T cell aggregation or "clustering" in control, anti-CD3/CD28-stimulated CXCR4^low^ CD4 T cells, and anti-CD3/CD28-stimulated CXCR4^hi^ CD4 T cells. Scale bar, 100μm. (F) The number of T cell aggregates or "clusters" in control, stimulated T derived from *CXCR4*^low^ T, and stimulated T derived from *CXCR4*^hi^ T. Data were shown as mean ± SEM.

The expression level of *CXCR4* has strong cell heterogeneity; e.g., *CXCR4* is highly expressed in most resting T cell subsets, while it is lowly expressed in most stimulated T cell subsets (Figure 4A). In particular, HSP^hi^ T highly expresses *CXCR4*, which is similar to resting T subsets and different from the other stimulated T cell subsets (Figure 4A). These findings are consistent with aforementioned observations that the gene expression profile of HSP^hi^ T is similar to that of T_N_. Considering the expression heterogeneity of *CXCR4*, we sorted resting CD4 T cells into *CXCR4*^*hi*^ T and *CXCR4*^low^ T by FACS (Figure 4B). Both *CXCR4*^hi^ T and *CXCR4*^low^ T were co-stimulated by CD3 antibody and CD28 antibody for 16 h. FACS analyses showed anti-CD3/CD28 co-stimulated CXCR4^low^ T expressed much higher level of CD25 than co-stimulated *CXCR4*^hi^ T (Figures 4C and 4D). Further analyses showed that the number of T cell aggregates or "clusters" in stimulated *CXCR4*^low^ T cells was significantly higher than that in stimulated *CXCR4*^hi^ T (Figures 4E and 4F). These results indicated that *CXCR4*^low^ T cells responded to anti-CD3/CD28 stimulation much more efficiently than *CXCR4*^hi^ T.

### Stimulated CD4 T cells showing different T cell subsets in the presence of CD8 T cells

**Figure 5:**
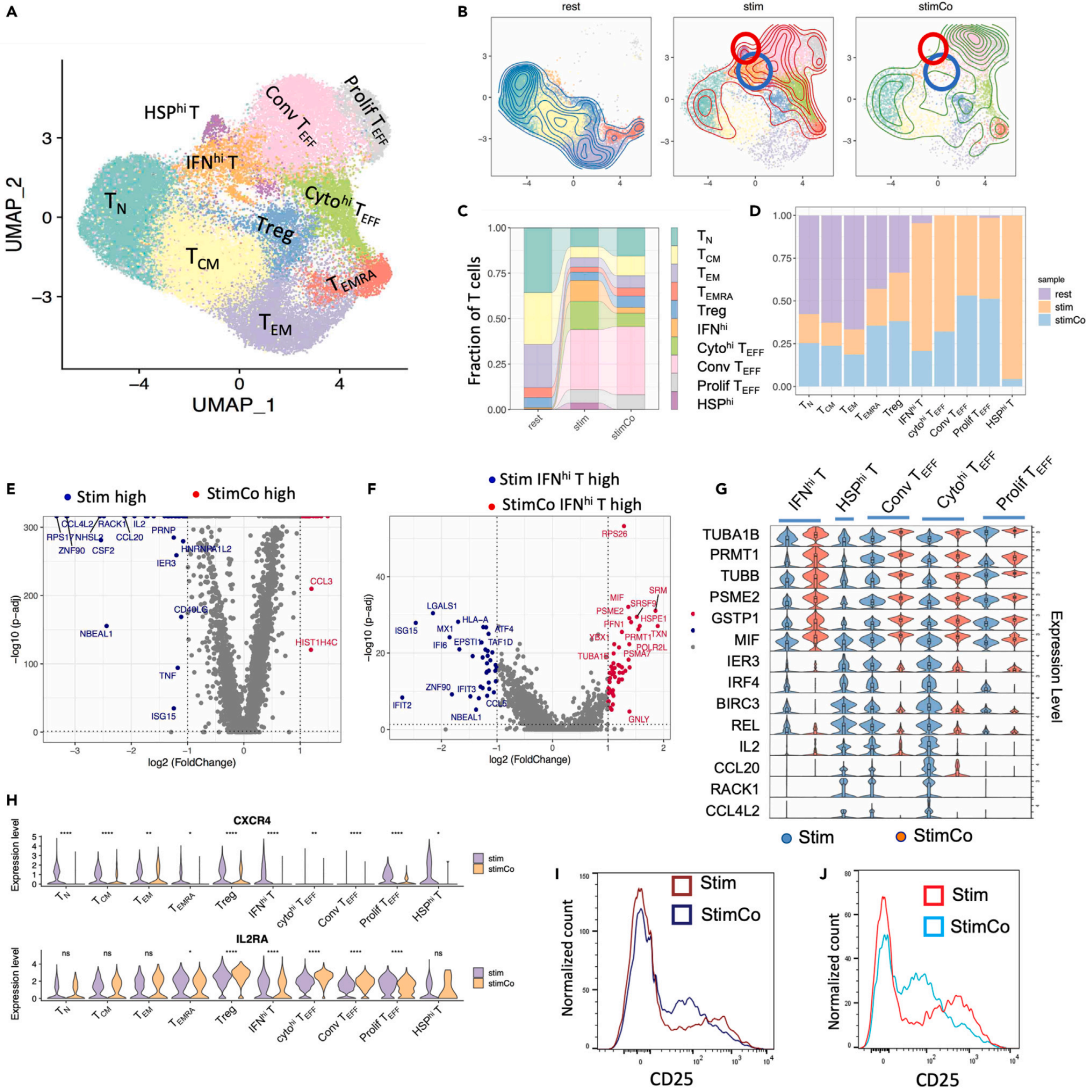
Changes of stimulated CD4 T subsets and signals when stimulations co-incubated with CD8 T (A) UMAP projection of CD4 T cells including rest T, stim T, and stimCo T, colored by T cell subset. (B) Differences of cell distributions of rest T, stim T, and stimCo T, with HSP^hi^ T and IFN^hi^ T marked by circles. (C) Fraction of T subsets in rest T, stim T, and stimCo T. The lines between two bars link the T cell subset counterparts in different samples. (D) Cell composition of T cell subsets based on cell sources. (E) DEGs between stim T and stimCo T. Also see Data D4. (F) DEGs of IFN^hi^ between stim T and stimCo T. (G) Expression level of stim T-specific genes and stimCo T-specific genes in activated T cell subsets. (H) Expression levels of *CXCR4* and *IL2RA* in T cell subsets of stim T and stimCo T. (I) FACS analyses of CD25 in stim T and stimCo T. (J) FACS analyses of CD25 in stimulated T derived from CXCR4^low^ T and stimulated T derived from CXCR4^low^ T in the presence of CD8 T. Also see Figures S4 and S5.

CD3 T cells, including CD4 T cells and CD8 T cells, also are commonly used model to study T cell activation.^35^ We performed scRNA-seq on both resting CD3 T cells and stimulated CD3 T cells. Analyses of CD3 T cells identified nine CD4 T subsets and seven CD8 T subsets (Figures S4A–S4E). Pseudotime analyses of CD3 T cells identified two major trajectories, which were CD4 T cell activation lineage and CD8 T cell activation lineage (Figure S4F), indicating activation of CD4 T cells and activation of CD8 T cells are two distinct events. In this way, we regard the CD4 T cells in stimulated CD3 T cells as stimulated CD4 T cells co-incubating with CD8 T cells (stimCo T). By integrated analyses of stimulated CD4 T cells (stim T) and stimCo T, we identified 10 CD4 T cell subsets, which essentially consist with the T cell subsets in Figure 1 (Figures 5A and S5A). A T cell subset, which highly expressed *ISG15*, *IFIT2*, *MX1*, *IFIT3*, *ISG20*, and *IFI44L*, and enriched GO terms such as defense response to virus (p = 6.31 × 10^−15^) (Figures S5B and S5C), was called IFN^hi^ T.

We found that stim T and stimCo T had different distribution and different density on UMAP plot (Figure 5B), indicating CD4 T cell activation was affected by the presence of CD8 T cells. In particular, stimCo T has higher density on conv T_EFF_ and much lower fraction on HSP^hi^ T and IFN^hi^ T than stim T (Figure 5B). Quantitatively, stim T has 32.9% conv T_EFF_ and 15.6% cyto^hi^ T_EFF_, while stimCo T has 37.4% conv T_EFF_ and 7.4% cyto^hi^ T_EFF_, indicating stimCo T have increased conv T_EFF_ and decreased cyto^hi^ T_EFF_ compared with stim T (Figure 5C). HSP^hi^ T and IFN^hi^ T account for 0.43% and 6.5% of StimCo T, respectively, which were much lower than their counterparts in stim T (4.92% HSP^hi^ T and 16.1% IFN^hi^ T) (Figure 5C). These findings indicate that stimulation of CD4 T with CD8 co-incubation might promote CD4 T differentiation into conv T_EFF_ while repress CD4 T differentiation into HSP^hi^ T, IFN^hi^ T, and cyto^hi^ T_EFF_. Indeed, HSP^hi^ T, IFN^hi^ T, and cyto^hi^ T_EFF_ are mainly from stim T, with few contributions from stimCo T (Figure 5D).

### Different signals of T cell activation between stim T and stimCo T

We identified a total of 56 significantly DEGs between stim T and stimCo T, including 21 stimCo T-specific genes and 35 stim T-specific genes (Data S4). The 21 stimCo T-specific genes including *TUBB*, *TUBA1B*, *MIF*, *GSTP1*, *SRM*, and *HIST1H4C*, many of which involved in regulation of protein kinase and response to stimulus, indicating stimCo T have strong effector function (Figure 5E). The 35 stim T-specific genes are significantly enriched in cytokine signaling (*CCL4*, *IL2*, *CCL20, CCL4L2, TNF, ISG15*) (Figure 5E). We further identified the DEGs between stim T subset and stimCo T subset in each T cell subset. Stim IFN^hi^ T highly expressed genes related to IL-mediated signaling (*MX1, OAS1, OASL, IFIT3*), while stimCo IFN^hi^ T highly expressed genes related to profilin binding and actin and tubulin folding (*ACTB, TUBA1B, DNAJA1, HSPA8*) (Figure 5F). Stim conv T_EFF_ highly expressed cytokine genes (*CD40LG, CSF2, IFNG, IL2, IRF1, CCL20*), while stimCo T_EFF_ highly expressed genes related to detoxification of reactive oxygen species (*LTA, GSTP1, PRDX2, TXN, CCL3, LCK*) (Figure S5D). Overall, stim T subsets highly expressed cytokines or stimulation response genes (*CCL4L2*, *RACK1*, *CCL20, IL2, REL, BIRC3, IRF4,* and *IER3*) (Figure 5G), while stimCo T subsets highly expressed signal transduction and cellular functional genes (*TUBA1B, PRMT1, TUBB, PSME2, GSTP1, MIF*) (Figure 5G). Pseudotime analyses showed that trajectory of stimCo T extended and differentiated sufficiently, while the trajectory of stim T terminated earlier, with a large number of cells being in the midway of the trajectory (Figures S5E–S5G). Therefore, these results support that stimulation of CD4 T cells in the presence of CD8 T cells significantly increases the immunological functions of activated T while reducing the over-response and cytokine production.

Stim T and stimCo T have similar activation level since the stimulations were conducted in the same experimental conditions.For example, the expression level of *IL2RA*, a representative T cell activation marker, in stimCo T is similar to that in stim T (Figure 5H). On the other hand, we found that stimCo T has a higher proportion of conv T_EFF_ and expresses effector function-associated genes higher than stim T (Figures 5A–5G). Furthermore, most stimCo T cell subsets express *CXCR4* significantly lower than stim T cell subsets (Figure 5H), potentially indicating that stimCo T responds to stimulation more efficiently and more homogeneously than stim T. We re-conducted T cell stimulation and analyzed the heterogeneities of stim T and stimCo T by FACS. FACS analysis showed distribution of CD25 expression in stim T was broader and with more extreme values than that in stimCo T (Figure 5I), supporting that stimCo T is more homogeneous than stim T. We further sorted and stimulated *CXCR4*^low^ T with/without the presence of CD8 T. Distribution of CD25 expression in stimulated T derived from *CXCR4*^low^ T was much broader than their counterpart in the presence of CD8 T cells (Figure 5J), further supporting that the presence of CD8 T made the response of CD4 T become more homogeneous. These results potentially indicate that CD8 T could significantly change the stimulated T cell subsets and expression signals by cell-cell communication or its secreting cytokines.

### CD8 T-initiated cytokine signaling and FAS/FASLG signaling influenced CD4 T cell activation

**Figure 6:**
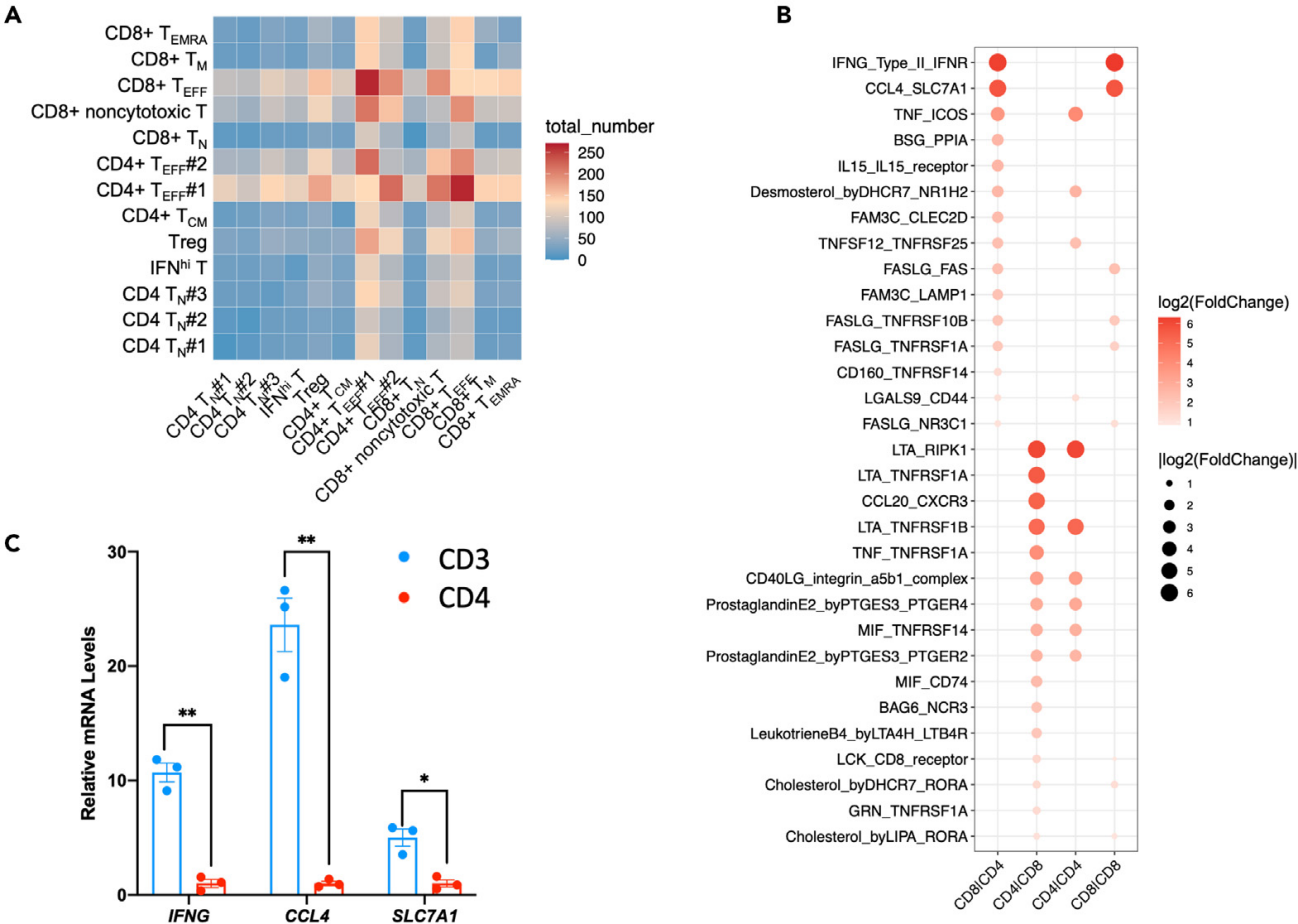
The interaction between CD8 T and CD4 T in simulated CD3 T cells (A) Heatmap of cell-cell communication strength between T cell subsets in simulated CD3 T cells. (B) Dot plot of the interaction between ligands and receptors among CD8 T and CD4 T. (C) Expression of *IFNG*, *CCL4*, and *SLC7A1* in stimulated CD3 T cells and stimulated CD4 T cells, measured by qPCR. Data were shown as mean ± SEM.

In order to determine how CD8 T affects CD4 T cell activation, we analyzed cell-cell communication between CD8 T and CD4 T in stimulated CD3 T using CellPhoneDB.^36^ We found cell-cell communication between CD4 T_EFF_ subsets and CD8 T_EFF_ subsets was much stronger than that between other T cell subsets in stimulated CD3 T (Figure 6A). We found CD8 T cells initiate the cytokine signaling, including IFNG_Type_II_IFNR, CCL4_SLC7A1, IL15_IL15_receptor, TNF_ICOS, FAM3C_CLEC2D, and FAM3C_LAMP1 (Figure 6B), which potentially enhance the immune response or immune function of CD4 T cells. On the other hand, CD8 T cells also initiate FAS/FASLG signaling, including FASLG_FAS, FASLG_TNFRSF10B, FASLG_TNFRSF1A, and FASLG_NR3C1 (Figure 6B), which is essential for immune system regulation such as activation-induced cell death and cytotoxic T lymphocyte-induced cell death. In fact, analyses of the expression of *IFNG, CCL4*, and *SLC7A1* in stimulated CD3 T cells and stimulated CD4 T cells showed these genes were CD3 T-specific expression (Figure 6C), supporting that CD8 T initiate these cytokines signaling and FAS/FASLG signaling. Therefore, CD8 T cells affect CD4 T cells mainly by cytokine signaling and FAS/FASLG signaling, which changed the CD4 T cell activation process. In particular, FAS/FASLG signaling is crucial for immune system regulation, which explains why the presence of CD8 T promotes CD4 T differentiation into conv T_EFF_ while repressing CD4 T differentiation into HSP^hi^ T, IFN^hi^ T, and cyto^hi^ T_EFF_. FAS/FASLG signaling may also explain why the presence of CD8 T made the stimulated CD4 T more homogeneous.

### The effects of different factors on CD4 T cell activation

**Figure 7:**
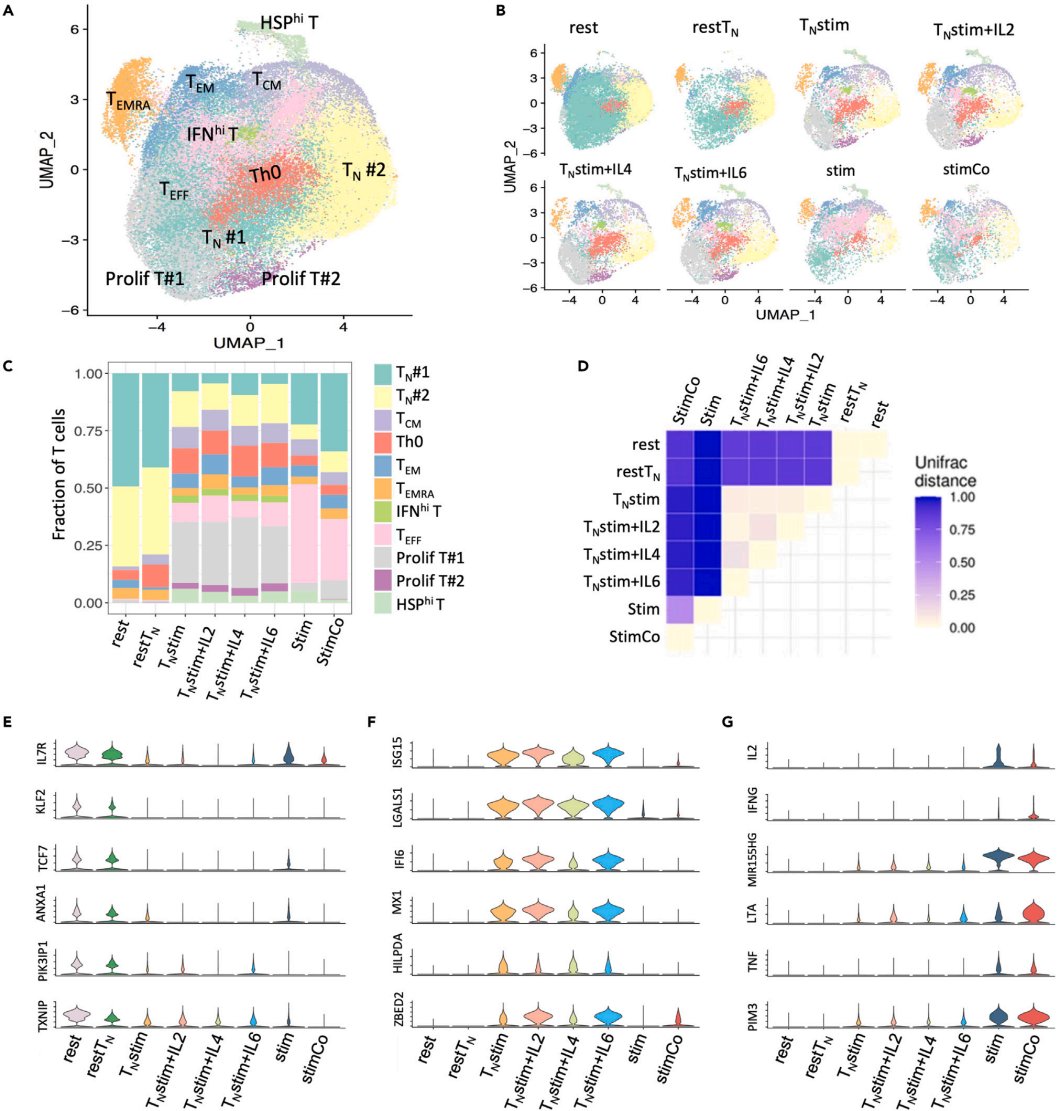
Exploring multiple factors affecting CD4 T cell activation by integrating 8 samples (A) UMAP projection of integrated CD4 T cells from 8 conditions, colored by cell subset. (B) UMAP projection of CD4 T cells in each condition, namely, rest, restT_N_, T_N_stim, T_N_Stim+IL2, T_N_Stim+IL-4, T_N_Stim+IL6, stim, and stimCo. (C) Bar plot of fraction of T cell subsets in each condition. (D) UniFrac distances between CD4 T cell samples summarized in a correlation plot. The values of UniFrac distance range from 0 to 1, with lower value indicating higher similarity. (E–G) Expression levels of rest mode-specific genes (E), T_N_stim mode-specific genes, (F) and stim mode-specific genes (G) in each sample. Also see Figure S6.

To explore the factors affecting CD4 T cell activation, we further integrated scRNA-seq data of naive CD4 T cells (restT_N_) and stimulated CD4 T cells derived from CD4 T_N_ (T_N_stim) and T_N_stim in the presence of different cytokine cocktails.^20^ The integrated 5 datasets are restT_N_, T_N_stim, T_N_stim in the presence of IL2 and transforming growth factor β (TGF-β) (T_N_stim+IL2), T_N_stim in the presence of IL-4 and anti-IFNγ (T_N_stim+IL-4), and T_N_stim in the presence of IL6, IL23, IL1β, TGFβ, anti-IFNγ, and anti-IL-4 (T_N_stim+IL6). The integrated 60,639 CD4 T cells were re-clustered into 11 T cell subsets, including 5 resting T cell subsets (T_N_ #1, T_N_ #2, T_CM_, T_EM_ and T_EMRA_) and 6 activated T subsets (IFN^hi^ T, T_EFF_, Th0, prolif T #1, prolif T #2, and HSP^hi^ T) (Figures 7A and S6A). The cell composition of the 8 samples displayed three distinct modes: “rest mode” representing resting T cells (rest and restT_N_), “T_N_stim mode” representing stimulated T derived from T_N_ (T_N_stim, T_N_stim+IL2, T_N_stim+IL-4, T_N_stim+IL6), and “stim mode” representing stimulated T derived from peripheral CD4 T (stim and stimCo) (Figures 7B and 7C). Majority of the cells in rest mode are T_N_ #1 and T_N_ #2, which is quite different from stim mode and T_N_stim mode (Figures 7B and 7C). Stim mode has the highest fraction of T_EFF_ among all the three modes (Figure 7C). Compared with stim mode, T_N_stim mode has increased proportions of Prolif T, ISAG^hi^ T, HSP^hi^ T, and Th0.

We further calculated UniFrac distance^37^ to quantify the similarity of cell composition between two samples. UniFrac distance ranges from 0 to 1, where 0 indicates two samples have the same cell composition and 1 indicates two samples have entirely separate clusters. UniFrac distances between two samples within each mode are much smaller than those across modes, supporting that intra-mode differences are much weaker compared to the inter-model differences (Figure 7D). In particular, UniFrac distances between T_N_stim mode and stim mode are high, indicating that type of resting T cells is the dominant factor affecting T cell activation (Figure 7D). While the UniFrac distance between stim and stimCo is the highest among all within-mode comparisons, indicating co-incubation of CD8 T is the second strongest factor affecting T cell activation (Figure 7D). Indeed, stimCo T displays a distinct distribution on the UMAP compared to the other stim or T_N_stim (Figure S6B). Samples in rest mode highly expressed resting T cell-specific genes such as *TCF7*, *KLF2*, *ANXA1*, *TXNIP*, and *IL7RA* (Figure 7E). Samples in T_N_stim mode highly expressed IFN-associated genes such as *ISG15, MX1*, and *IFI6* (Figure 7F), consistent with our observation that these samples have the highest fraction of IFN^hi^ T. Samples in T_N_Stim mode also highly expressed *LGALS1*, *ZBED2*, and *HILPDA* that promote cell proliferation,^38^ consistent with pronounced increase of prolif T. Samples in stim mode highly expressed *PIM3, TNF, LTA, MIR155HG, IFNG*, and *IL2* (Figure 7G), consistent with the pronounced increase of conv T_EFF_ in these samples.

## Discussion

CD4 T cells are one of the most commonly used models for studying T cell activation. Most of the previous studies focused on CD4 T_N_ cells because they are relatively homogeneous^4^ and can differentiate into various functional T cell subsets.^8^^,^^20^ In order to analyze the factors affecting T cell activation, we chose the most easily obtainable CD4 T cells, peripheral CD4 T cells, to initiate the study. Although the peripheral CD4 T cells are highly heterogeneous, including CD4 T_N_, CD4 T_CM_, CD4 T_EM_, CD4 T_EMRA_, and ISAG^hi^ T,^4^^,^^35^ we still could investigate the signals of T cell activation because the analyses were conducted at single-cell level. Among the 4 T_EFF_ subsets generated after stimulation, cyto^hi^ T_EFF_ expressed the highest level of *IL2* and *IFNG*, indicating cyto^hi^ T_EFF_ has high fraction of mainly Th1. However, we did not separate the CD4 T_EFF_ into different T helper subsets because we focused on the genome-wide expression profile. Among the stimulated CD4 T cells, HSP^hi^ T enriched HSP genes and genes related to cellular responses to stress. HSP^hi^ T were mainly projected on T_N_ when we re-performed UMAP projection using genes without HSP genes, indicating HSP^hi^ T have an expression profile similar to T_N_ except highly expressed HSP genes. We also identified a stimulated T cell subset that highly expressed IFN-associated genes, namely IFN^hi^ T. In short, resting CD4 T cells mainly differentiate into different T_EFF_ upon anti-CD3/CD28 stimulation, while a small fraction of CD4 T cells differentiate into specific T cell subsets such as HSP^hi^ T and IFN^hi^ T.

The existence of inert T indicates some resting T cells respond to stimulation much slower than the other T cells due to the heterogeneity of resting CD4 T cells. The inert T has an expression profile similar to resting T, indicating the features of inert T are similar to those of resting T. However, inert T expressed activated T-specific genes such as *IL2RA*, *NME1*, *MIR155HG*, *FABP5, IL2, CD200*, and *IER3*, indicating inert T are not complete quiescent and have responded to the anti-CD3/CD28 stimulation. Furthermore, the activated T-specific genes were intermediately expressed in inert T, supporting that inert T is a transitional state between resting T and activated T. On the other hand, inert T cells highly expressed cytokine genes (*CCL3, CCL4, CCL4L2, CCL3L3, TXNIP, CD52, CXCR4*, and *CORO1A*) compared with activated T and resting T, indicating inert T is not a simple transition state with weak effect function but with its own special feature. For example, the expression level of *CXCR4* decreases successively in resting T, inert T, and activated T, while HSP^hi^ T is an outlier in the activated T cells since it highly expresses *CXCR4*. We found the expression profile of HSP^hi^ T was similar to that of T_N_ if we did not consider the HSP genes, indicating HSP^hi^ T was mainly derived from stimulated T_N_.

Comparison of stimulated T cells under several conditions showed that UniFrac distance between stimulated peripheral CD4 T and stimulated CD4 T_N_ was the highest among all comparisons. In this way, the type of resting T cells has the strongest effects on stimulated T cells. Indeed, stimulation of peripheral CD4 T and CD4 T_N_ resulted in significant differences of stimulated T cell subsets. In particular, stimulated T derived from peripheral CD4 T has much higher fraction of T_EFF_ while stimulated T derived from CD4 T_N_ has much higher fraction of prolif T. The second important factor affecting T cell activation is whether the stimulation was co-incubated with CD8 T cells. Our analysis showed CD4 T cells were more likely to differentiate into T_EFF_ and were less likely to differentiate into HSP^hi^ T and IFN^hi^ in the presence of CD8^+^ T cells. The effects of CD8 T cells co-incubation on T cell activation may be mediated by cell-cell communications and/or specific microenvironment shaped by their secreted cytokines. Finally, presence of cytokine cocktail during stimulation also shaped the T cell subsets in some way but does not have strong effects based on their expression profiles.

In summary, this study was the first endeavor that systemically explored the factors affecting T cell activation at single-cell resolution. First, we characterized inert T which is a transitional state during differentiation of resting T into T_EFF_. Second, we found and characterized HSP^hi^ T, a stimulated T subset highly expressing HSPs, mainly derived from stimulated naive T (T_N_). Third, we found the stimulation of CD4 in the presence of CD8 significantly reduced the HSP^hi^ T and INF^hi^ T and increased T_EFF_ in the stimulated T. These differences could be explained by CD8 T-initiated cytokine signaling and FAS/FASLG signaling that could regulate CD4 T cell activation.

### Limitations of the study

The current study provided insights into T cell dynamics and the factors influencing T cell activation, while there are some limitations to consider. First, the study focused on *in vitro* stimulation of T cells using anti-CD3/CD28 antibodies, which may not fully mimic the *in vivo* environment. Second, the study has identified unique subsets during T cell activation, such as the HSP^hi^ T cells, but lacks functional validation. Additional experiments and functional assays using animal model may enhance the robustness and comprehensiveness of the findings. Finally, the study uncovered the influence of CD8 T cells on shaping CD4 T cell activation profiles; further investigation and experimental validation are warranted to dissect the precise mechanisms and signaling involved.

## STAR★Methods

### Key resources table

**Table:** 

REAGENT or RESOURCE	SOURCE	IDENTIFIER
**Antibodies**		

CD25-PE (clone: BC96)	eBioscience	Cat #12-0259-42; RRID: AB_1659682
CXCR4-APC (clone: 12G5)	Biolegend	Cat #306510; RRID: AB_314616
Dynabeads™ Human T-activator CD3/CD28	ThermoFisher	Cat #11131D; RRID: AB_2916088
CD3 MicroBeads, human	Miltenyi Botec	Cat #130-050-101; RRID: AB_2910233
CD4 MicroBeads, human	Miltenyi Botec	Cat #130-045-101; RRID: AB_2889919

**Chemicals, peptides, and recombinant proteins**		

Lymphoprep	STEMCELL	Cat #07851
TRIzol	Invitrogen	Cat #15596026

**Critical commercial assays**		

Chromium™ Single Cell 3′ Library & Gel Bead Kit v2, 16 rxns	10x Genomics	Cat #PN-120237
ReverTra Ace™ qPCR RT Kit	TOYOBO	Cat #FSQ-101
SYBR Green Real-time PCR Master Mix	TOYOBO	Cat #QPK-201

**Deposited data**		

Raw and processed scRNA-seq data	This paper	HRA002777
Code	This paper	https://github.com/JinWLab/T_active

**Oligonucleotides**		

Human IFNG Forward:5′-TCGGTAACTGACTTGAATGTCCA-3′	PrimerBank	PrimerBank ID: 56786137c1
Human IFNG Reverse:5′-TCGCTTCCCTGTTTTAGCTGC-3′	PrimerBank	PrimerBank ID: 56786137c1
Human CCL4 Forward:5′- CTGTGCTGATCCCAGTGAATC-3′	PrimerBank	PrimerBank ID: 4506845a1
Human CCL4 Reverse:5′- TCAGTTCAGTTCCAGGTCATACA-3′	PrimerBank	PrimerBank ID: 4506845a1
Human SLC7A1 Forward:5′- GCCTGTGCTATGGCGAGTTT-3′	PrimerBank	PrimerBank ID: 209571525c2
Human SLC7A1 Reverse:5′- ACGCTTGAAGTACCGATGATGTA-3′	PrimerBank	PrimerBank ID: 209571525c2

**Software and algorithms**		

Cell Ranger v3.0	10x Genomics	https://github.com/10XGenomics/cellranger
Seurat v4	Stuart et al., 2019	https://satijalab.org/seurat/index.html
Metascape	Tripathi et al., 2015	http://metascape.org
Monocle3	Trapnell et al., 2014	https://cole-trapnell-lab.github.io/monocle3/
GraphPad 9	GraphPad	https://www.graphpad.com
FlowJo v10	FlowJo LLC	https://www.flowjo.com/

### Resource availability

#### Lead contact

Further information and requests for resources and reagents should be directed to and will be fulfilled by the lead contact, Wenfei Jin (jinwf@sustech.edu.cn).

#### Materials availability

This study did not generate new unique reagents.

### Experimental model and study participant details

#### Healthy donors

This study was approved by IRB at Southern University of Science and Technology (SUSTech). The human peripheral blood samples used in this study were obtained from two healthy adult donor: one Chinese male (age: 40 years) and one Chinese female (35 years). For the scRNA-seq, peripheral blood sample from the male donor was used. For FACS analysis and qPCR experiments, peripheral blood samples from the male and the female donor were used. All experiments were conducted following the protocols approved by IRB at SUSTech. Informed consent was obtained from the two participants before sample collection.

### Method details

#### Isolation of peripheral blood mononuclear cells (PBMCs)

This study was approved by IRB at Southern University of Science and Technology (SUSTech). The human peripheral blood samples used in this study were obtained from two healthy adult donors. All experiments were conducted following the protocols approved by IRB at SUSTech. PBMCs were isolated by density-gradient sedimentation of peripheral blood using Lymphoprep (STEMCELL, Cat #07851, Germany) following previous study.^4^ Briefly, peripheral blood sample was diluted with an equal volume of PBS containing 2% FBS, transferred into a Lymphoprep tube. Following centrifuged at 800 × g for 20 min at room temperature, the middle white phase was transferred to a fresh tube. The enriched mononuclear cells were washed with PBS containing 2% FBS and centrifuged at 500 × g for 5 min twice.

#### Isolation of CD3 T cells and CD4^+^ T cells

The CD3^+^ cells and CD4 T cells were enriched from PBMCs using CD3 MicroBeads (Miltenyi Biotec, #130-050-101, Germany) and CD4 MicroBeads (Miltenyi Biotec #130-045-101, German), respectively, following products’ instructions. In brief, PBMCs were re-suspended and washed with PBS buffer, and magnetically labeled with CD3 MicroBeads or CD4 MicroBeads. Then the cell suspension was loaded onto a MACS Column in the magnetic field of a MACS Separator. The magnetically labeled CD3^+^ cells or CD4^+^ T cells were retained on the column. After removal of the column from the magnetic field, the magnetically retained CD3^+^ cells or CD4^+^ cells were collected by elution.

#### T cell activation by anti-CD3/CD28 stimulation

MACS enriched CD4^+^ T cells and CD3^+^ T cells were stimulated with Dynabeads Human T-Activator CD3/CD28 for T cell Expansion and Activation (ThermoFisher, Cat# 11131D, USA) following the manufacturer’s instructions. Briefly, 8 × 10^4^ CD4^+^ T cells were suspended with 100–200 μL medium. Pre-washed Dynabeads was added at the bead-to-cell ratio of 1:1 and incubated in a humidified CO_2_ incubator at 37°C. After 16 h, stimulated CD4^+^ T cells were harvested by removal of Dynabeads, and then stained with anti-CD25 (eBioscience, Cat #12-0259-42, USA) for FACS analyses of the efficiency of T cell activation. The CD3 T+ cells were stimulated with Dynabeads Human T-Activator CD3/CD28 for T cell Expansion and Activation following exactly the same protocol as stimulation of CD4^+^ T cells.

#### scRNA-seq library preparation and sequencing

MACS enriched CD4^+^ T cells (resting CD4 T cells) and anti-CD3/CD28 stimulated CD4^+^ T cells (stimulated CD4 T cells) were directly processed for scRNA-seq with Chromium Single Cell 3′ Library & Gel Bead Kit v2 (10x Genomics, Pleasanton, CA) following manufacturer’s instructions. For each sample, cells were counted in a haemocytometer chamber and about 1.6 × 10^4^ T cells were loaded into single inlet of a 10x Genomics Chromium controller to generate the nanoliter-sized Gel beads in Emulsions (GEMs). After GEM collection, cell lysis, RNA-capture and barcoded reverse transcription were performed inside each GEM, thus mRNA was reverse transcribed into cDNA. After breaking the oil droplets, libraries were generated from the cDNAs with Chromium Single Cell 3′ Library Construction Kit v2 (PN-120237, 10x Genomics). Sequencing libraries were loaded at 2.4 p.m. into Illumina NovaSeq 6000 with 2 × 150 paired-end kits using the following read length: 28 bp Read1, 8 bp I7 Index, 8 bp I5 Index and 91 bp Read2.

#### Integration of public data and data pre-processing

We generated the scRNA-seq data of resting CD4 T cells, resting CD3 T cells, anti-CD3/CD28 stimulated CD4 T cells, anti-CD3/CD28 stimulated CD3 T cells, and biological replicates. In order to systematically analyze the factors affecting T cell activation, we integrated public scRNA-seq datasets including scRNA-seq data of Naive CD4 T cells and diversified anti-CD3/anti-CD28 stimulated CD4 T cells from Cano-Gamez,^20^ scRNA-seq data of resting CD4 T cells (resting_2) and anti-CD3/CD28 stimulated CD4^+^ T cells (stimulated_2) from Ding,^39^ and scRNA-seq data of resting CD3 T cells (CD3 resting_2) from Massoni-Badosa.^40^

We mapped reads to reference genome (hg19) and generated single-cell gene expression matrix for each scRNA-seq dataset using Cell Ranger (v3.0). We used Seurat^41^ for quality control and basic pre-processing following our previous studies.^4^^,^^42^^,^^43^ We filtered out the cells with the following criteria: 1) number of detected genes <200; 2) UMI counts >40,000; 3) mitochondrial percentage >7.5%,^44^ 4) percentage of CD8 and erythrocyte gene >0.01%.

#### Dimension reduction and visualization of scRNA-seq data

Data integration, data normalization, dimension n reduction and visualization were performed using Seurat,^41^ similar to our previous studies.^4^^,^^42^^,^^43^ We identified the top 3,000 high variable genes using *FindVariableFeatures* function and further scaled the gene expression matrix using *ScaleData* function. We conducted principal component analysis (PCA) for linear dimension reduction using *RunPCA* function and the top 30 PCs were used for further analysis. We visualized cells using Uniform Manifold Approximation and Projection (UMAP).^45^ Clusters were divided by an unsupervised graph-based clustering method.

#### Identification of cell subsets and differential gene analysis

The cell clusters were annotated using T cell subset specific gene markers from reference articles. In order to identify the differentially expressed genes between two subsets or two conditions, we utilized function FindMarkers to exert differential expression analysis on cluster pairs. It returned average log Fold Change (avg_logFC) and adjusted p value (p_val_adj) for each gene. The genes with p_val_adj <0.05 and avg_logFC>1 were treated as the differentially expressed genes (DEGs).

#### Gene Ontology (GO) enrichment analysis

In order to better understand the functions of cluster specific genes or DEGs between two T cell subsets, GO enrichment analyses were conducted on these gene lists using Metascape (http://metascape.org).^46^

#### Pseudotime inference

Monocle3^47^ uses pseudotime to measures how a cell moves through biological progress. We used monocle3 to infer trajectories of T cell activation on UMAP space and identified trajectory-associated differential genes. Naive T cells were selected as root node manually based on biological knowledge. The top 10 trajectory lineage associated genes were selected by the morans_I index.

#### Comparison of T cell activation between CXCR4^hi^ T and CXCR4 ^low^ T

In order to analyze whether the expression level of *CXCR4* affecting T cell activation, CD4 T cells or CD3 T cells were sorted into *CXCR4*^hi^ T cells and *CXCR4*^low^ T on FACS Aria-III (Becton Dickinson) with anti-CXCR4 (Cat # 306510, Biolegend). FACS sorted *CXCR4*^hi^ T cells and *CXCR4*^low^ T cells were co-stimulated with anti-CD3 antibody and anti-CD28 antibody. Briefly, 8 × 10^4^ FACS sorted *CXCR4*^hi^ T cells or *CXCR4*^low^ T cells were suspended with 100–200 μL medium, respectively. The anti-CD3 and anti-CD28 antibodies were added at the antibody-to-cell ratio of 1:1 and incubated in a humidified CO_2_ incubator at 37°C. After 16 h, activated T cells were harvested and stained with anti-CD25 (eBioscience, Cat #12-0259-42, USA) for FACS analyses of the efficiency of T cell activation. FlowJo (version 10) was used for FACS data analyses and Figure plot.

#### Quantitative reverse transcription polymerase chain reaction (RT-qPCR)

Gene expression levels were quantified using RT-qPCR assay. Approximately 0.5–1 million cells were collected for RNA extraction. Total RNA was extracted using TRIzol (Invitrogen, Cat #15596026, USA) according to the manufacturer’s instructions. Subsequently, around 2 μg of total RNA was used to synthesize cDNA using the ReverTra Ace qPCR Kit (TOYOBO, Cat #FSQ-101, Japan) following to the manufacture’s instructions. For RT-qPCR, the SYBR Green Real-time PCR master mix (TOYOBO, Cat #QPK-201, Japan) in a final volume of 20 μL. The PCR reactions were performed on an ABI StepOne Plus PCR system. The cycling conditions consisted of an initial denaturation at 95°C for 60s, followed by 40 cycles of denaturation at 95°C for 60s, annealing at 60°C for 15s, and extension at 72°C for 45s. Expression level of genes of interested were normalized by the expression level of *GAPDH*. All primers are listed in the key resources table.

### Quantification and statistical analysis

Data were shown as mean ± SEM. Two-tailed Mann-Whitney U-test was used to compare the statistical significance between two groups. p ≤ 0.05 was considered as significant. statistics were calculated with GraphPad Prism 9 (GraphPad software).

## Data Availability

• The single cell RNA-seq data has been deposited in Genome Sequence Archive in BIG Data Center. Accession number is HRA002777, with others in key resources table. • The original code of this paper is available at Github (https://github.com/JinWLab/T_active). • Any additional information required to reanalyze the data in this paper is available from the lead contact upon request.

## Supplemental information

Document S1. Figures S1–S6

Data S1. HSPhi T-specific genes comparing with other T cells, related to Figure 1D

Data S2. DEGs between inert T and resting T, related to Figure 3

Data S3. DEGs between inert T and activated T, related to Figure 3

Data S4. DEGs between stim T and stimCo T, related to Figure 5D
