# Supplementary material for: Exploring the dynamics and influencing factors of CD4 T cell activation using single-cell RNA-seq
Source: iScience. 2023 Aug 9;26(9):107588. doi: 10.1016/j.isci.2023.107588 (PMC10460988; doi:10.1016/j.isci.2023.107588)
Supplement: Document S1. Figures S1–S6 [file mmc1.pdf]

iScience, Volume *26*

Supplemental information

Exploring the dynamics and influencing factors of CD4 T cell activation using single-cell RNA-seq

Hui Li, Hongyi Liu, Yifei Liu, Xuefei Wang, Shiya Yu, Hongwen Huang, Xiangru Shen, Qi Zhang, Ni Hong, and Wenfei Jin

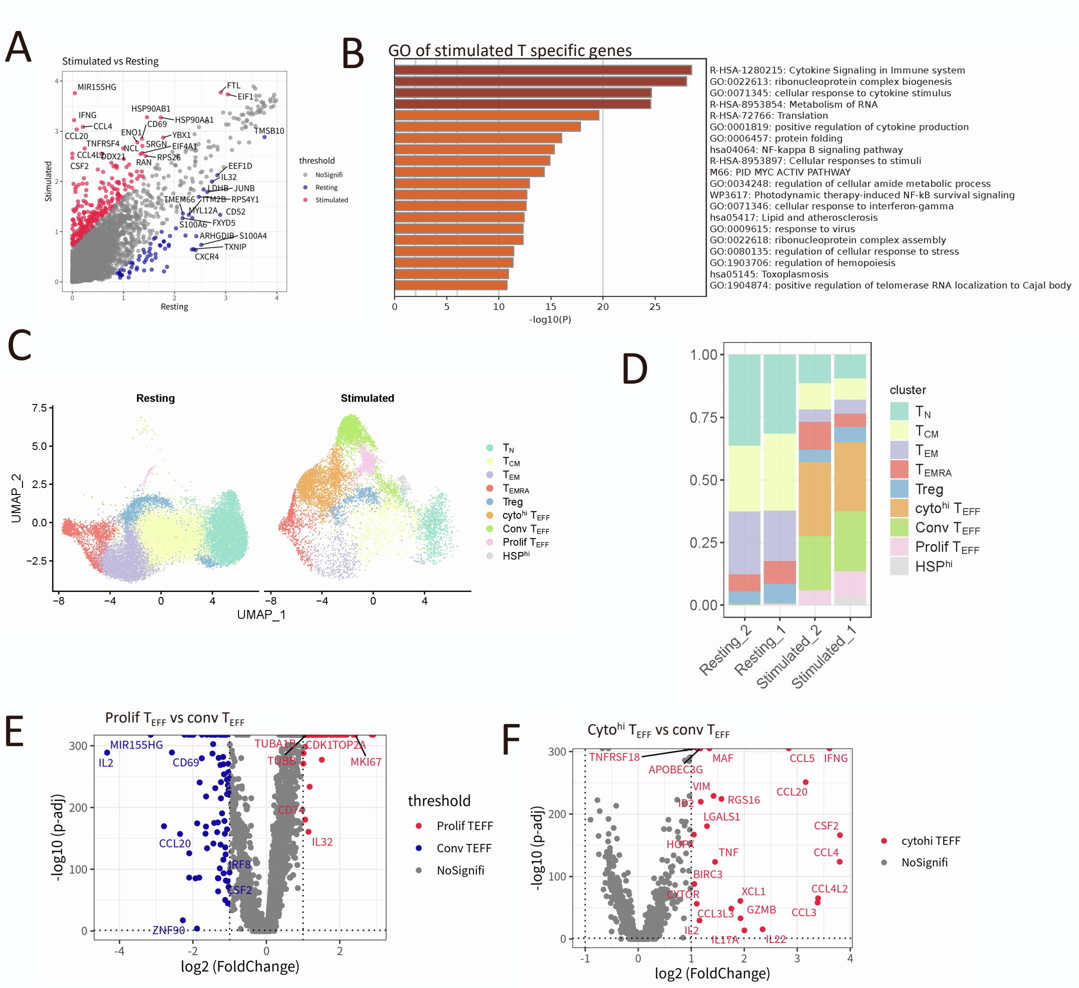

Fig. S1. Feature and subsets of CD4 T cell pre-and post-stimulation. (A) Scatter plots of gene expression level in resting CD4+ T cells and stimulated CD4+ T cells. Blue dot and red dot represent resting T specific genes and stimulated T specific genes, respectively. **(B)** GO enrichment analysis of stimulated CD4 T cell specific genes. **(C)** UMAP visualization of resting CD4 T cells and stimulated CD4 T cells, colored by cell subset. **(D)** Fraction of T subsets in each resting T samples and stimulated T samples. **(E)** Volcano plot of DEGs between conv T_{EFF} and prolif T_{EFF}. Red points represent prolif T_{EFF} specific expressed genes, while blue points represent conv T_{EFF} specific expressed genes. **(F)** Volcano plot of DEGs between conv T_{EFF} and cyto^{hi} T_{EFF}. Red points represent cyto^{hi} T_{EFF} specific expressed genes. **Related to Fig. 1.**

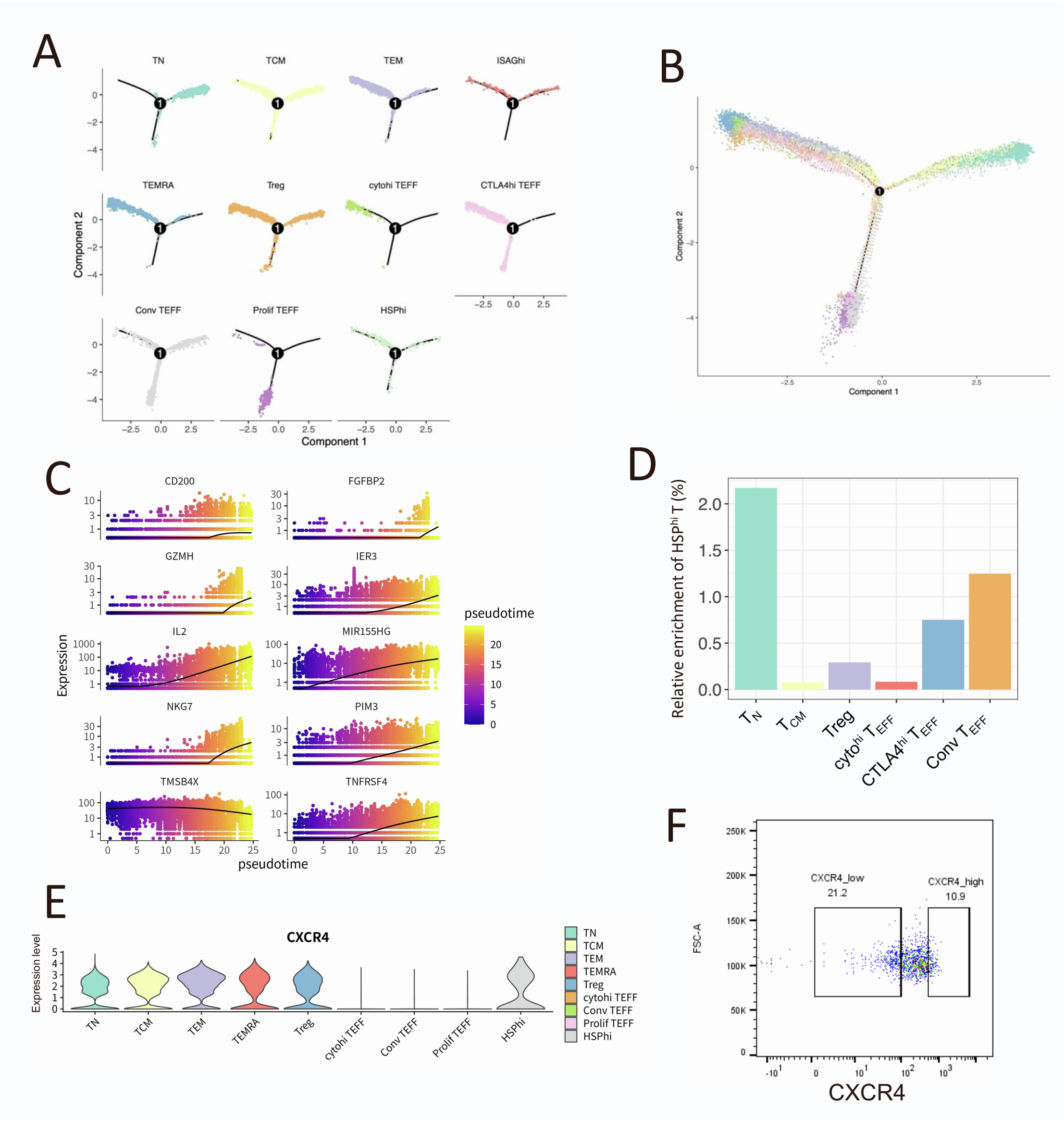

Fig. S2. Pseudotime trajectory of T cell activation and trajectory associated genes. (A-B) Ordering of cells along pseudotime in two-dimensional state space defined by Monocle2. Cells are colored by cellular clusters. Individual cluster projections **(A)** and overlay of all clusters **(B). (C)** Genes associated with T cell activation trajectory inferred by Monocle3. **(D)** Bar plot of number of HSP^{hi} T normalized to its projected T cell subsets. **(E)** Expression level of *CXCR4* in each T cell subset. **(F)** Enrichment of HSP^{hi} T cells (CXCR4^{high}) and CXCR4^{low} T cells by FACS. **Related to Fig. 2.**

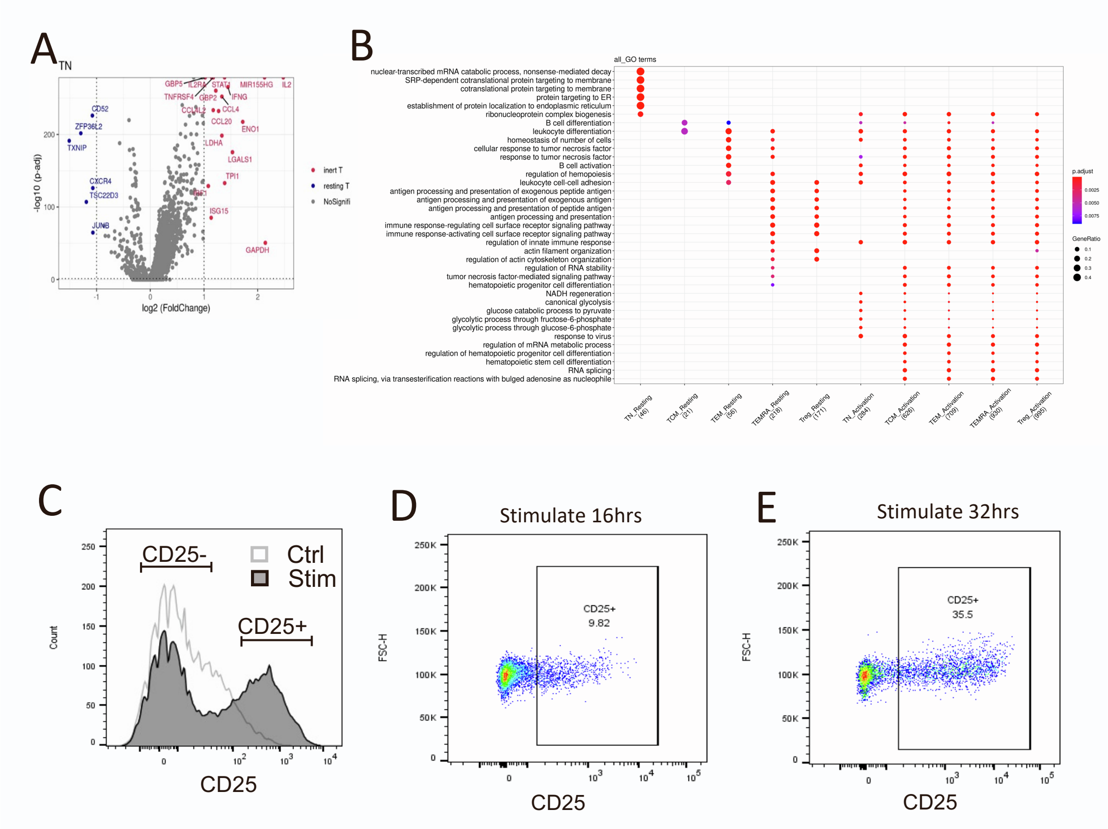

Fig. S3. Feature and subsets of inert T and its dynamics. (A) Volcano plot of DEGs between inert T_N and resting T_N. Red point and blue point represent inert T_N specific expressed genes and resting T_N specific expressed genes, respectively. (**B**) GO analysis of inert T subset specific genes and resting T subset specific genes (avg_logFC >= 0.25) showing the enriched GO terms of inert T subset specific genes were shared. The color of the dot represented the adjustive p value, and the size of the dot represent-ed the ratio of expressing genes counts to all genes counts. **(C)** FACS analysis of CD25 expression level after activation of CD4 T cells. (**D-E**) FACS analysis of CD25 in stimulated T cells after 16 hours (D) and 32hours (E). **Related to Fig. 3.**

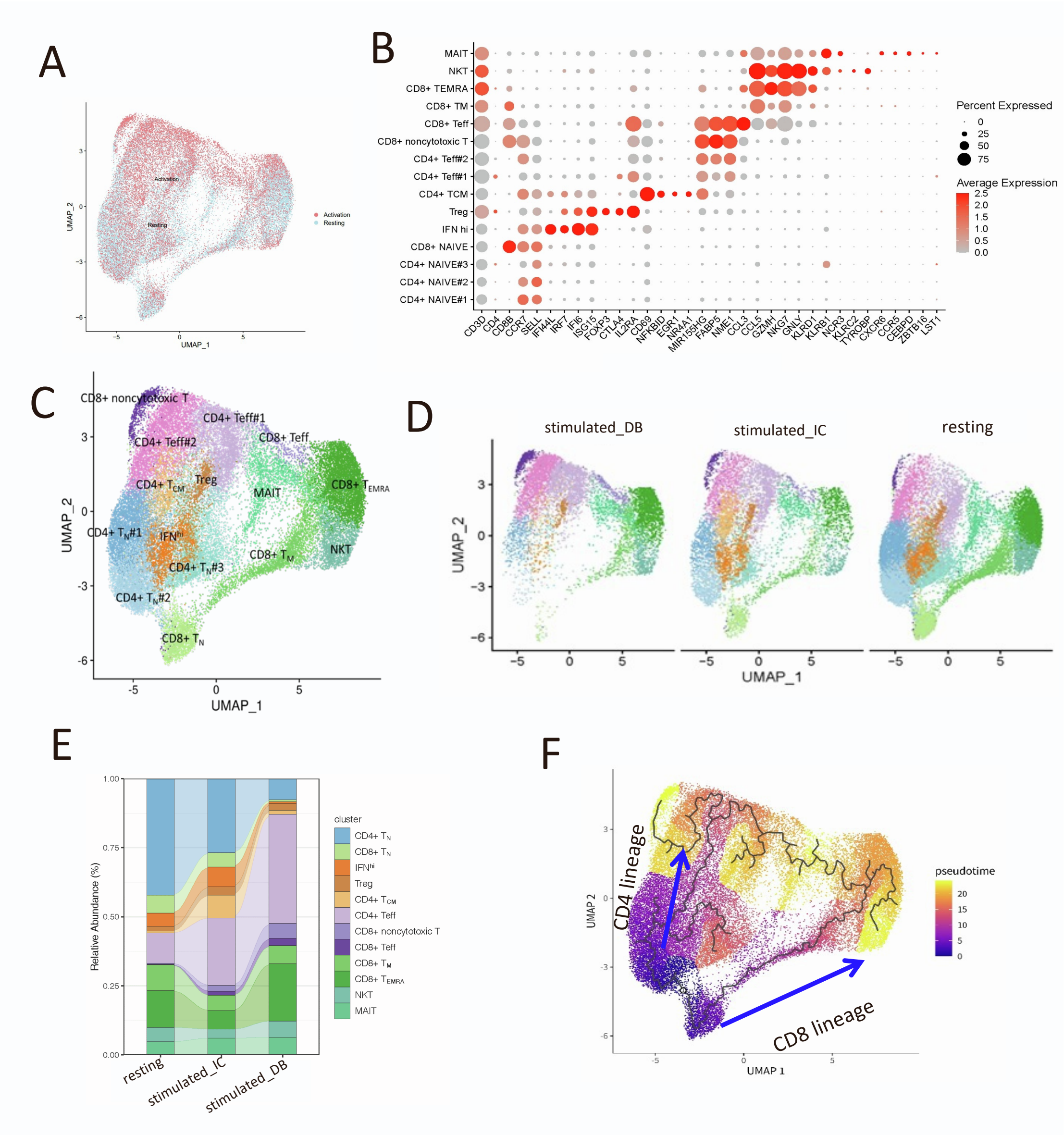

Fig. S4. T cell subsets and lineage of CD3 T cell activation. (A) UMAP projection of CD3 T cells pre-and post-stimulation, colored by whether it was stimulated. **(B)** Normalized expression level and expression percentage of the cell-type-specific genes in the 15 T cell subsets. Color represents normalized expression level, and dot size represents expression percentage. **(C)** UMAP projection of CD3+ T cells pre-and post-stimulation, colored by T cell subset. **(D)** UMAP Projection of resting T cells, stimulated T cells by ImmunoCultTM (stimulated_IC) and stimulated T cells by DynabeadsTM (stimulated_DB), colored by cell subset. **(E)** Fraction of T cell subsets in resting T cells, stimulated_IC, and stimulated_DB. The lines between two bars link the T cell subset counterparts in different samples. **(F)** Two distinct lineages, namely CD4+ T lineage and CD8+ T lineage, were displayed in CD3 T cell activation process. **Related to Fig. 5.**

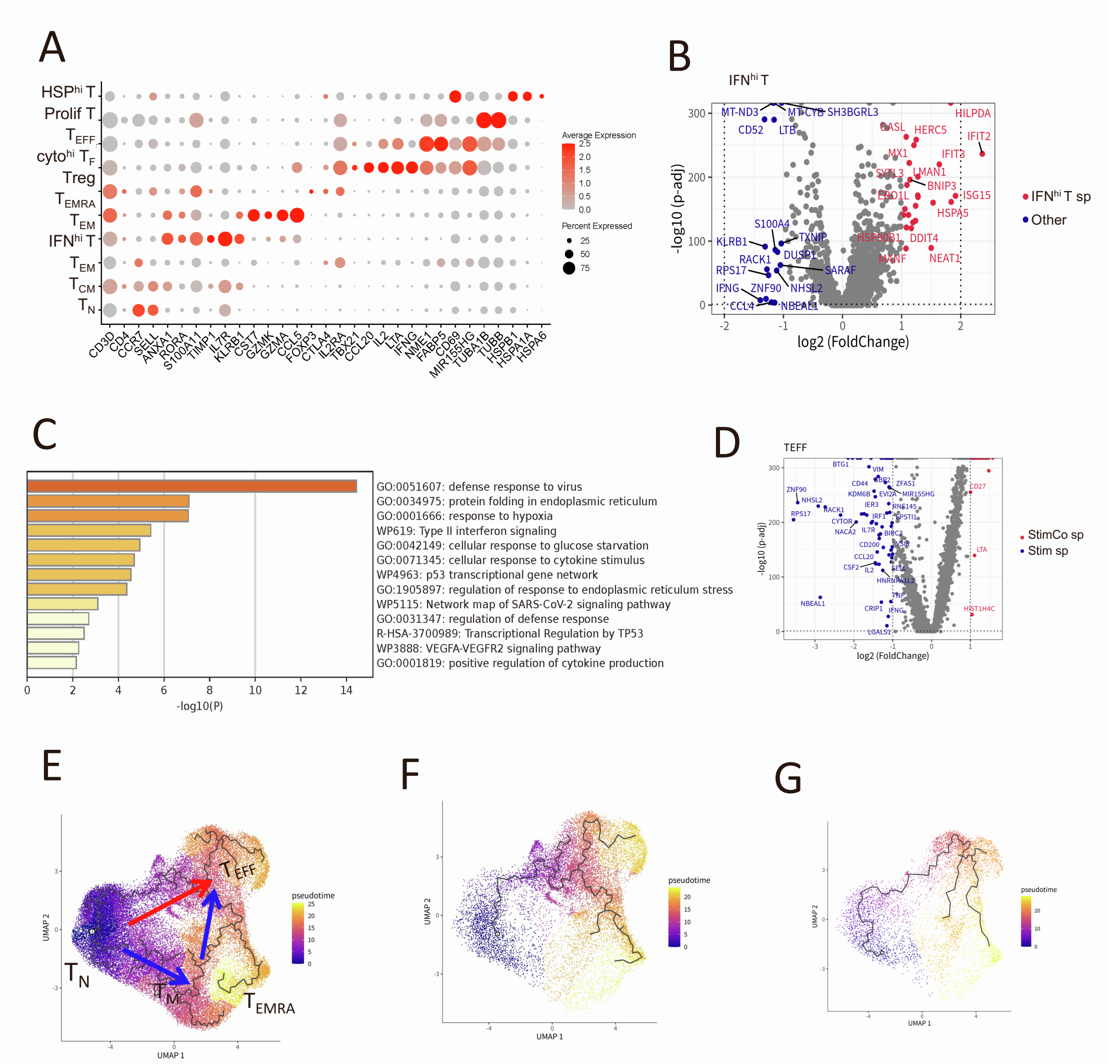

Fig. S5. Integrated stimulated CD4 T cells co-incubated with CD8 T cells. (A) Normalized expression level and expression percentage of cell-type-specific genes in the 10 subsets. **(B)** Volcano plot of DEGs between IFN^{hi} T and other T cells. **(C)** GO analysis of IFN^{hi} T specific genes. **(D)** Volcano plot of DEGs between stimCo T_{EFF} and stim T_{EFF}. **(E)** Inferred two main lineages in stimulated CD4+ T cells: T_N->T_{EFF}; T_N->T_M->T_{EFF}. **(F)** Inferred lineage in stim T. **(G)** Inferred lineage in stimCo T. **Related to Fig. 5.**

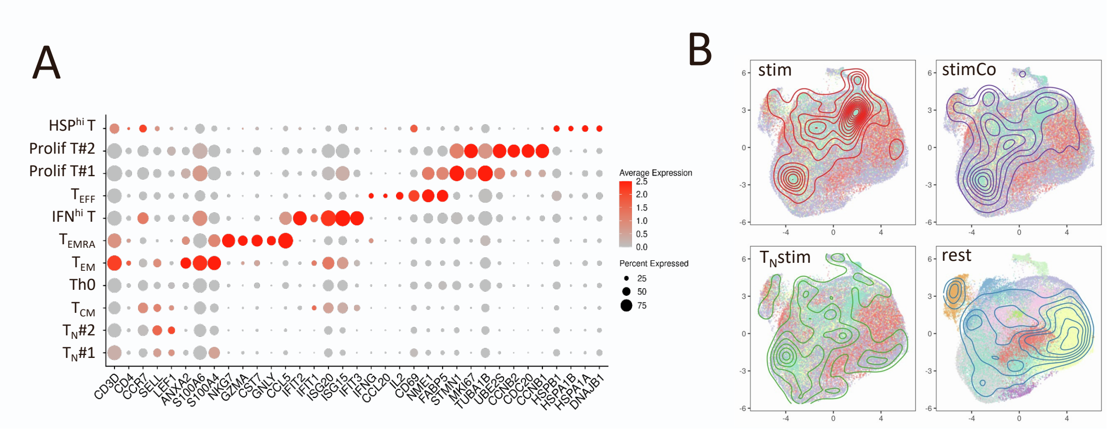

Fig. S6. Analyses of CD4 T cell activation with multiple samples. (A) Normalized expression level and expression percentage of cell-type-specific genes of the 11 subsets in integrated T cells. **(B)** UMAP projection of the CD4 T cells with 4 major conditions, namely rest, stim, stimCo, and $T_N$stim. **Related to Fig. 7.**
